# Single-Cell Informatics for Tumor Microenvironment and Immunotherapy

**DOI:** 10.3390/ijms25084485

**Published:** 2024-04-19

**Authors:** Jiabao Tian, Xinyu Bai, Camelia Quek

**Affiliations:** Faculty of Medicine and Health, The University of Sydney, Sydney, NSW 2006, Australia; jtia5428@uni.sydney.edu.au (J.T.); xbai6546@uni.sydney.edu.au (X.B.)

**Keywords:** cancer, cancer environment, tumor immune communication, bioinformatics, treatment response, immunotherapy

## Abstract

Cancer comprises malignant cells surrounded by the tumor microenvironment (TME), a dynamic ecosystem composed of heterogeneous cell populations that exert unique influences on tumor development. The immune community within the TME plays a substantial role in tumorigenesis and tumor evolution. The innate and adaptive immune cells “talk” to the tumor through ligand–receptor interactions and signaling molecules, forming a complex communication network to influence the cellular and molecular basis of cancer. Such intricate intratumoral immune composition and interactions foster the application of immunotherapies, which empower the immune system against cancer to elicit durable long-term responses in cancer patients. Single-cell technologies have allowed for the dissection and characterization of the TME to an unprecedented level, while recent advancements in bioinformatics tools have expanded the horizon and depth of high-dimensional single-cell data analysis. This review will unravel the intertwined networks between malignancy and immunity, explore the utilization of computational tools for a deeper understanding of tumor–immune communications, and discuss the application of these approaches to aid in diagnosis or treatment decision making in the clinical setting, as well as the current challenges faced by the researchers with their potential future improvements.

## 1. Introduction

**Figure 1:**
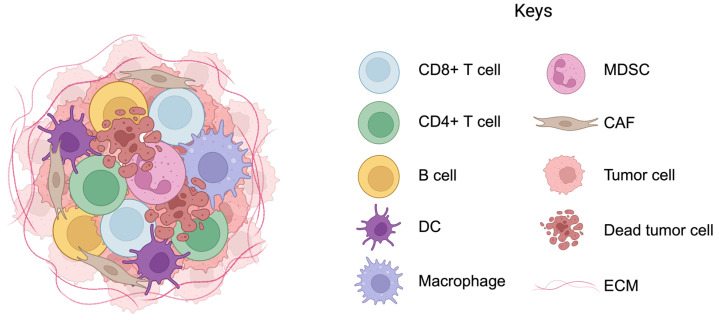
A basic schematic view of the tumor microenvironment (TME) showing different cell types. Cancer cells are surrounded by a wide diversity of innate and adaptive immune cells. The coordination between the immune and tumor cells regulates homeostasis and initiates an appropriate immune response to cancer. The complex network of cells within the TME supports and influences tumor growth and response to cancer therapies. Abbreviations: DC—dendritic cell; MDSC—myeloid-derived suppressor cell; CAF—cancer-associated fibroblast; ECM—extracellular matrix.

The tumor microenvironment (TME) is a complex and dynamic ecosystem comprising immune and stromal compartments. As shown in Figure 1, the immune compartment consists of diverse cell types that suppress or stimulate tumor progression [1,2,3,4,5]. To suppress tumor growth, antitumorigenic populations, such as the T and B lymphocytes, directly target and eliminate the tumor cells or activate the antitumor capacity of other immune cells, such as macrophages [6,7,8,9]. On the contrary, pro-tumorigenic communities, including the myeloid-derived suppressor cells (MDSCs) and M2 macrophages, exert immunosuppressive effects through the inhibition of antitumor functions and the promotion of tumor escape from immunosurveillance [10,11].

Cell–cell coordination within the TME is critical for initiating appropriate antitumor immune responses. The coordination networks are established through cell–cell communications (CCC), predominantly through ligand–receptor (LR) interactions at the cell-surface level. Cells “talk” to one another by using the “key” ligand to “unlock” the receptor, initiating a cascade of downstream signaling events [12]. For example, the interaction between programmed death-ligand 1 (PD-L1) on tumor cells and programmed cell death protein 1 (PD-1) expressed by CD8+ T cells induces T cell exhaustion, downregulating its antitumor cytotoxicity and promoting tumor growth [13]. Meanwhile, the release of signaling cytokines and chemokines promotes cellular communications. Interferon γ released by cytotoxic CD8+ T cells is a classic example that initiates tumor apoptosis or necrosis and promotes cancer degradation [14].

Immune checkpoint inhibitors (ICIs), a type of immunotherapy that harnesses immunity against tumors, have revolutionized cancer treatment. The most investigated checkpoint inhibitors are cytotoxic T lymphocyte antigen-4 (CTLA-4) and PD-1 monoclonal antibodies. CTLA-4, an inhibitory receptor commonly present on naïve CD4+ T cells, binds to CD80/86 ligands with high affinity. Such binding downregulates T cell priming, thus inhibiting cellular activation and proliferation [8,15]. Anti-CTLA-4 blocks CTLA-4 functions, enabling CD80/86 to engage with the co-stimulatory molecule CD28, thereby promoting T cell activation [16]. Likewise, persistent PD-L1/PD-1 binding between tumor and CD8+ T cells turns the latter into exhaustion, diminishing its cytotoxicity against tumor antigens [17]. Anti-PD-1 interrupts the PD-L1/PD-1 interaction to recover the cytotoxic capacity of CD8+ T cells and enhance tumor elimination [13,18]. However, ICI therapies have not yet achieved optimal efficacy owing to the distinct response rates observed across different cancers, ranging from 4% for pancreatic cancer to 70% for melanoma [19]. A comprehensive understanding of cancer biology, particularly regarding the cellular composition of the TME and the tumor–immune cell cross-talk, is urgently required to improve patient response to ICI therapies.

Traditional bulk analyses have unraveled genetic changes that substantially modulate tumor evolution and antitumor immunity, significantly enhancing our understanding of the intrinsic and extrinsic factors that drive tumor growth. However, the average gene expressions measured across numerous cells obscure the nuances between rare subpopulations and cellular states, masking crucial biological details associated with disease development and treatment response. Moreover, traditional proteomic assessments such as immunohistochemistry, in situ hybridization, and flow cytometry rely on predefined panels featuring limited antibodies, constraining their abilities to comprehensively and accurately depict the cellular and molecular basis of cancer. Recent advances in single-cell technologies enable whole-transcriptome sequencing at single-cell resolution. The tremendous amount of data generated potentiates our understanding of tumor biology [20,21,22,23]. Meanwhile, subsequent developments in bioinformatics tools facilitate the precise characterization and visualization of the TME, enabling efficient and straightforward interpretation of massive and complex results. Among these tools, transcriptomic analyses are highly mature, with over 1400 bioinformatics approaches currently available [24]. Such advancement has dramatically improved the identification of gene markers to aid in defining and discriminating patient heterogeneity and varied treatment responses.

**Figure 2:**
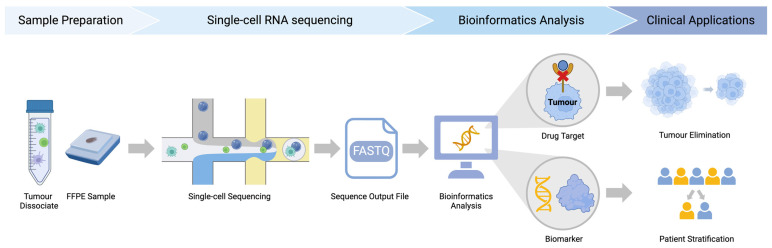
A flowchart demonstrating the critical steps that are sequentially performed in single-cell technologies. Different types of tumor samples, including tumor dissociates or formalin-fixed paraffin-embedded specimens, undergo preparation. Subsequently, these samples are subjected to single-cell resolution sequencing using sequencing platforms, generating a FASTQ output file containing gene expression profiles of individual cells. These gene profiles then undergo various bioinformatics analyses to uncover biological insights, such as identifying drug targets and biomarkers, which are mainly used for shrinking tumor size and helping stratify patients who likely respond or resist the therapy. Abbreviations: FFPE—formalin-fixed paraffin-embedded.

Cancer research using single-cell technologies is a four-step process, starting from sample preparation and ending with clinical application (Figure 2). In this review, we summarize and discuss the widely applied step-based bioinformatics tools for characterizing cellular composition and predicting cancer–immune cell cross-talk, providing helpful guidance for study design and method selection. Moreover, we select key studies that associate biological discoveries with therapy outcomes to demonstrate the translational significance of bioinformatics results in clinical practices. We present the current challenges faced by these methods while suggesting future directions to spark the advancement of novel bioinformatics approaches, broadening their utility across a spectrum of diseases. Publications from the past five years are the main focus of this review. Studies regarding bioinformatics tools were selected based on their wide application in single-cell data analysis; tumor-related articles were selected based on their high relevance to the subject matter.

## 2. Single-Cell Technologies

Single-cell technologies largely offset the limitations of bulk analyses by capturing the subtle differences between cell types, refining our understanding of cancer and its interactions with the TME (Table 1). This section provides an overview of single-cell approaches in the aspects of gene sequencing and cellular spatial imaging, demonstrating significant improvements in measuring analyte availability, amplifying throughput, and improving detection resolution.

### 2.1. Single-Cell Sequencing

Single-cell RNA sequencing (scRNA-seq) workflows are primarily categorized into plate-based or droplet-based methods. The plate-based SMART-seq2 and MARS-seq use conventional fluorescence-activated cell sorting (FACS) to isolate each cell to a well on a multi-well plate [31,32]. Sequencing of full-length transcripts is allowed this way, making it particularly useful for detecting rare cell types and low-abundance genes. However, the plate-based approach significantly increases workload as only a limited number of cells (up to hundreds) can be simultaneously processed. On the contrary, droplet-based methods, including 10x Chromium, Drop-seq, and inDrop, spare the use of FACS by encapsulating cell suspension into individual partitions, in which single cells are isolated and tagged [33,34]. This method not only provides a more accessible entrance and shorter processing time but also exhibits high throughput, as thousands of cells are processed within a single batch. Nevertheless, detecting only 3′ or 5′ transcripts may disadvantage their ability to identify differentially expressed genes. Therefore, the choice of technology should be made based on specific study objectives.

### 2.2. Spatial Single-Cell Technologies

Recent advancements in spatial omics technology allow a larger number of markers to be detected in spatial contexts. Current approaches use either fluorophore-tagged antibodies to detect protein markers at cellular levels (imaging-based) or RNA probes to recognize mRNA transcripts at transcriptomic levels (next-generation sequencing-based [NGS-based]). Imaging-based approaches such as ChipCytometry, CyCIF, and MICs involve iterative cycles of antibody staining, imaging, and stripping using a customized multiplexed panel [35,36,37]. Since the panel is typically restricted to 30–50 antibodies and the results are relatively qualitative, complementary genomic information using the NGS-based approaches is required for validation. MERFISH, SeqFISH, Slide-seqV2, and Visium HD enable the detection of up to 10,000 transcripts from which quantitative results are generated, significantly addressing the shortage of using antibody-based methods alone [38,39,40]. Notably, methods including PhenoCycler (CODEX), DBiT-seq, CosMx, and Xenium enable the simultaneous detection of both protein and RNA markers while allowing for the integration of transcriptomic and proteomic data, leading to a more comprehensive interpretation and visualization of the spatial results [41,42,43,44].

## 3. Bioinformatics Tools for Single-Cell Data Analysis

### 3.1. Bioinformatics Dissection of Tumor–Immune Cell Microenvironment

Single-cell technologies have helped us better understand the complexity and dynamics of the TME. The massive amount of data generated by scRNA-seq poses challenges to downstream analysis due to the complex high-dimensional data structure and intensive workload. Bioinformatics analysis helps extract biologically relevant information from high-dimensional data through data preprocessing, normalization, confounder correction, dimensionality reduction, clustering, and annotation (as summarized in Table 2). Standardized pipelines such as Seurat [45], Scanpy [46], and Bioconductor-based SingleCellExperiment [47] provide comprehensive guidance for each step, while additional approaches are recommended to accomplish peripheral analysis tasks.

#### 3.1.1. Quality Control

Quality control (QC) is the first critical step in data preprocessing. QC aims to remove ambient RNA contamination and poor-quality cells, preserving only high-quality cells for downstream analysis. Ambient RNAs are biologically irrelevant cell-specific transcripts in the cell suspension. If not removed, they are likely misidentified in other cell types, obscuring the distinction between different cell populations during the clustering step [50]. SoupX [50] and CellBender [51] are able to address the problem by estimating and removing the cell-free gene markers in a supervised and unsupervised manner, respectively. Moreover, scDblFinder [53], a Bioconductor-based package that detects and eliminates doublets or multiplets, helps remove low-quality cells. It has recently been examined as the best performer among the currently available doublet-detecting tools [83].

#### 3.1.2. Gene Count Normalization

The gene counts in high-quality cells must undergo normalization to minimize technical variations introduced during the experimental process. The most popular normalization approach is global scaling, whereby each cell is calculated with a “size factor” based on which the gene expressions are scaled to a standard reference, making the expressions comparable between cells [84]. Analysis pipelines, such as Seurat, Scanpy, and BASiCS [59], have integrated normalization methods based on scaling, regression, and spike-in RNAs, respectively [57,59,85,86,87,88]. The recently compiled regression-based method sctransform is becoming more prevalent. It omits the heuristic steps while treating the sequencing depth as a covariate, producing more objective and robust results with lower false positive rates [55].

#### 3.1.3. Confounder Correction

Correction of confounding factors is another necessary step involved in data cleaning. Technical confounders, such as batch effects, are corrected by scVI and scGen for the complex integration of multiple samples, or by Harmony for simple sample integrations [62,63,89]. In addition, biological confounding factors, such as cell cycle effects, may be directly modified by the built-in functions in Seurat or Scanpy, alongside methods such as Tricycle, if the dataset displays highly heterogenous cell populations [66,90].

#### 3.1.4. Feature Selection

This step ensures that only biologically informative gene features are selected and preserved for downstream analysis. Townes et al. examined and compared the three currently used approaches for feature selection—deviance, highly variable genes, and highly expressed genes—across eight scRNA-seq datasets. The effectiveness of deviance stood out mainly due to its ability to identify both highly expressed and highly variable genes [91]. Moreover, deviance-based approaches perform feature selection directly on the raw counts of unique molecular identifiers, making them insensitive to normalization methods [92].

#### 3.1.5. Dimensionality Reduction

The challenge of interpreting high-dimensional data with a sparse structure makes dimensionality reduction essential for downstream analysis. Dimensionality reduction simplifies the data by reducing the number of dimensions while preserving the essential structure and variations within the data. A common approach is the principal component analysis (PCA), which captures the maximal variances of the original variables, reducing the total dimensions to a few principal components [93]. However, the linear model used by PCA is unable to perfectly reflect the non-linear scRNA-seq structure, leading to the inevitable loss of biological variations during the reduction process. Comparisons between multiple approaches have revealed that the graph-based, non-linear methods such as t-distributed stochastic neighbor embedding (t-SNE) and uniform manifold approximation and projection (UMAP) exhibit higher robustness and accuracy, albeit they come with a high computational cost [94,95].

#### 3.1.6. Cell Clustering

Following data preprocessing, individual cells with similar genetic profiles are grouped into clusters to summarize and explain data heterogeneity. Louvain, the k-nearest neighbor graph-based community detection algorithm, was reported with optimal performance [96,97]. However, Traag et al. reported that the Louvain-based calculations resulted in poorly connected (25%) or even disconnected (16%) communities [98]. The Leiden algorithm was successively introduced, guaranteeing intercommunity connections while increasing the clustering speed.

#### 3.1.7. Annotation

Finally, the clusters are annotated with specific biological interpretations. The biggest concern impeding accurate annotation is the lack of a standardized protocol, leading to biased and subjective biological definitions that are highly inconsistent across different studies. Heumos and colleagues proposed a three-step approach consisting of automated annotation, manual annotation, and expert verification, aiming to minimize bias and increase the objectivity and validity of annotation [99]. Automated annotation is implemented using either the classifier-based methods, such as CellTypist [78] and Clustifyr [79], or the reference-based approaches, such as Azimuth [81] and SingleR [82]. Notably, the results generated by automated annotation heavily rely on the classifier type and the quality of training datasets, suggesting the importance of carefully aligning study goals with the chosen datasets. Meanwhile, clusters should be manually annotated by comparing the cluster-specific differentially expressed genes, identified via t-tests or Wilcoxon rank-sum tests, to the markers documented in well-referenced datasets. The final step is to seek verification from experts with solid immunology backgrounds, as their empirical knowledge may provide valuable insights into studies under specific contexts.

### 3.2. Bioinformatics Analysis of Tumor–Immune Cell Communication

Advancements in scRNA-seq technologies and the compilation of protein–protein interaction databases have enabled the inference and analysis of CCC. Currently used CCC inference tools utilize different computational strategies emphasizing specific biological aspects, including LR interaction, intracellular signaling, and spatial proximity [100]. Appropriate approaches for CCC analysis should be carefully considered depending on the data availability and overall study objectives. Here, we list the common bioinformatics approaches of CCC analysis alongside a brief explanation of their algorithms, strengths, and limitations.

#### 3.2.1. Ligand–Receptor Interaction

CCC inference largely relies on calculating the interaction strength, indicating the probability of interaction between a specific ligand and its receptor(s). The key LR-based bioinformatics approaches are listed in Table 3, with example studies and a brief explanation of the methods. Differential combination-based tools, such as iTALK [101], CellTalker [102], and PyMINEr [103], priorly compute the differentially expressed ligand- and receptor-encoding genes, which are compared to the reference databases to identify the LR pairs. However, since the differential genes are computed against the background gene expression, those with ubiquitously high expression are overlooked, thus potentially diminishing their valuable biological relevance. Another widely applied method is based on permutation that is integrated into platforms such as the CellPhoneDB [104], CellChat [105], ICELLNET [106], and SingleCellSignalR [107]. Ligand- and receptor-encoding genes are first selected and used to infer LR pairs, each calculated with a communication score informative of interaction strength. Significantly expressed LR interactions are selected based on multiple permutations of communication scores through non-parametric tests. False positive and negative rates are significantly reduced by this mean, making permutation the standard practice for CCC analysis and inference.

Notably, these inference tools exhibit few limitations, requiring cautious selection to minimize unexpected analysis results. Reference databases are curated based on the prior knowledge of LR interactions, overlooking the potential biological influence exerted by the novel interactions. Moreover, these platform-specific databases focus on different biological aspects, likely producing biased inference results even from analyzing the same dataset. For instance, the T cell receptor pathway was observed to be under-represented in most of the tools but was overrepresented in OmniPath and Celllinker [113]. Such discrepancy emphasizes the importance of strictly aligning the choice of platforms and databases with study objectives.

Moreover, iTALK and SingleCellSignalR infer cellular communication on the basis of single LR pairs, disregarding the influence of mediators or co-stimulatory molecules in initiating and promoting cell–cell interactions [101,102]. Recently compiled tools, such as CellChat and CellPhoneDB, mitigate this problem by using multiple LR pairs with the consideration of heteromeric complexes to increase inference power and reduce false positive rates [104,105]. Moreover, some may apply additional downstream analyses to extract further information. For instance, CellChat adopted the network centrality analysis that measures out-degree, in-degree, flow betweenness, and information centrality to uncover the highly specific roles of ligands and receptors on the directed weighted CCC networks. In consequence, this analysis unravels cellular components that mediate (mediator) or influence (influencer) LR interactions in addition to signal senders and receivers, providing deeper insights into the inferred cellular interactions and helping better understand their biological functions [105].

#### 3.2.2. Intracellular Signaling Communication

The subsequent signaling events following LR binding provide complementary information that validates the surface-level interactions. The integration of LR interactions and intracellular signaling thus greatly enhances the scope and accuracy for CCC inference at both protein and gene levels. Approaches that compute intracellular signaling, including NicheNet [114], scMLnet [115], CCCExplorer [116], scSeqComm [117], and CellCall [118], model interactions based on the changes occurring among ligands and/or receptors, transcription factors, and targeted genes, while others such as CytoTalk [119] measure the change in all intracellular genes.

#### 3.2.3. Spatial-Based Communication Inference

Recent advancements in spatial technologies at single-cell and subcellular resolutions enable the measurement of spatial proximity among individual cells. The trend of spatial-based CCC inference protocols increasingly prevails, given that physically proximal cells are more likely to interact. The built-in statistical methods are mainly based on two assumptions: Giotto [120], SpaOTsc [121], spaGCN [122], and DeepLinc [123] assume the co-existence of ligand and receptors for CCC occurrence, whereas SVCA [124] assumes that the gene expression of one cell relies on its interaction with the neighboring cells. Notably, while the development of either intracellular- or spatial-oriented CCC inference approaches is not mature enough for independent usage, combined strategies incorporating LR interactions are highly recommended to optimize inference accuracy.

#### 3.2.4. Experimental Validation of the Inferred CCC

The validation of predicted cell–cell interactions is crucial due to the probability-based nature of CCC inference. Laboratory experiments, such as functional assessments, examine the actual functions of the inferred ligands or receptors, while immunostaining enables the visualization of proteins on the cell surface, further confirming the occurrence of LR interactions [125]. The growing availability of spatial transcriptomic data offers another dimension that allows for the measurement of spatial distances between cell types to identify proximal cells, which are more likely to interact than those located far away [126]. However, this method may not be applicable to endocrine interactions, where distant cells communicate through traveling signals and hormones. Moreover, previously identified LR interactions serve as important validation sources. For instance, the presence of chemokine ligand 13 (CXCL13) on follicular helper T cells and chemokine receptor 5 (CXCR5) expressed by B cells strongly suggests their interaction in the cancer setting, supported by the previously identified CXCL13-CXCR5 chemotaxis in promoting cancer invasion and metastasis [127].

## 4. Application of Single-Cell Bioinformatics Tools in Understanding Patient Heterogeneity and Treatment Responses

Advancements in single-cell data analysis not only facilitate our understanding of tumor biology but also help establish the relationships between biological intricacies and clinical outcomes [128,129,130,131,132,133,134,135,136]. We have listed several studies that utilized single-cell technologies to explore the “How” and “Why” tumors respond differently to various treatments, with a focus on characterizing the diverse immune compartments, analyzing the tumor–immune cell interactions, and their roles in developing response or resistance to immunotherapies.

### 4.1. Utilizing Bioinformatics Analysis to Explore the Tumor Microenvironment (TME)

In the study on ICI-treated pancreatic ductal adenocarcinoma (PDAC) patients, Wang et al. used Seurat to compare the dense- to the loose-desmoplasia samples and identified eight major cell clusters, including tumor, immune, and cancer-associated fibroblast (CAF) cells [137]. Further dissection uncovered six subpopulations, among which the metabolic CAF (meCAF), a novel subtype with a high glycolysis rate, showed significantly high abundance in loose-type samples. High meCAF abundance positively correlates to metastasis and poor prognosis but is associated with an improved response to anti-PD-1, resulting in an ORR of 65%.

Another example utilized scRNA-seq and identified two cellular subtypes from both innate and adaptive immune systems. Team Krishna profiled samples from four ICI-treated patients with clear cell Renal Cell Carcinoma (ccRCC) and performed analysis via Seurat [138]. A positive correlation was identified between the pretreatment CD8+ tissue-resident T cell expression and patient response to ICI-based therapies. Moreover, the ISG^hi^ TAM subtype with elevated angiogenesis signatures was observed, evidenced by the increasing *FLT1*, *SPARC*, and *RGS5* expressions. Validated in multiple independent cohorts, ISG^hi^ TAMs are strongly associated with improved progression-free outcomes following sunitinib therapy.

Bioinformatics analysis extends beyond characterizing individual cell types to the computation of scoring schemes derived from multiple cellular states, enhancing the predictive power for heterogeneous treatment responses. For instance, scRNA-seq data derived from 35 non-small-cell lung cancer (NSCLC) samples were analyzed using Seurat, revealing 49 distinct immune populations [139]. Within the immune compartment, a high correlation among activated T cells, IgG+ plasma cells, and SPP1^hi^C1Q^lo^ monocyte-derived macrophages was identified and collectively computed to the lung cancer activation module (LCAM) score, greatly aiding in patient stratification. Validation of the LCAM score indicates that LCAM^hi^ patients exhibit better responses to anti-PD-1 treatment compared to those with a low LCAM score. The application of single-cell technologies unveils critical cellular phenotypes influencing treatment responses and prognosis, enhancing our understanding of tumor–immune cell interactions and predictive roles in tumor development and therapy response.

### 4.2. Application of Bioinformatics Analysis Regarding Tumor–Immune Cell Communication

Additionally, these studies employed single-cell technologies to unravel the cell–cell interactions, either to validate the single-cell results or directly correlate them with patient heterogeneity and therapy response. Wang et al. utilized CellChat to define tumor–immune cell cross-talks, revealing stronger interactions between the ECM matrix and dense-desmoplasia-specific PDAC cancer cells. These results underscore the crucial role of cellular communication in shaping tissue phenotypes and the TME structure [137]. Moreover, the co-existence of highly tumor-infiltrated ISG^hi^ TAMs and expanded CD8+ tumor-resident T cells in resistant patients suggests immunosuppressive effects exerted on the latter by the former. CellPhoneDB analyzed their interactions, revealing multiple suppressive interactions in between. The enhancement of T cell exclusion via TNFRSF1A-GRN and the polarization of TAMs toward immunosuppressive phenotypes via ADRB2-VEGFB highlight the complex interplay between immune populations. On the contrary, interactions promoting pro-inflammatory responses (e.g., CXCR3-CXCL9) were observed in patients with complete responses [138]. Furthermore, CellPhoneDB was employed to define LR interactions among immune cells in the LCAM-based NSCLC patient groups. In the LCAM^hi^ group, greater interactions were observed between T and B cells, particularly via CXCL13-CXCR5 and TNFRSF9-TNFSF9, to promote T cell activation. These strong interactions support the use of LCAM for patient stratification and shed light on the impact of tumor–immune cell cross-talk on shaping the LCAM axis [139].

## 5. Challenges and Future Directions

Single-cell technologies have developed to an unprecedented level, enabling detailed investigation into the diverse cellular composition and tumor–immune cell interactions. However, accurate sequence capture and analysis of high-dimensional data structures remain challenging. Here, we briefly discuss the major concerns regarding the use of single-cell technologies and bioinformatics analysis for TME characterization and CCC inference, with potential solutions to alleviate the problems. Furthermore, we propose the “bench-to-bedside” translational significance of the biological discoveries derived from single-cell technologies to provide valuable insights into its real-life applications in improving clinical outcomes.

### 5.1. Challenges Faced by TME Dissection via Single-Cell Technologies

One major challenge faced by scRNA-seq is the measurement of numerous “zero” gene expressions. These missing values are largely attributed to technical variations, such as degraded mRNA transcripts, inefficient transcript capture or amplification, or variable cell library dilutions, which greatly impede downstream analysis. Imputation helps solve such problems using model-based or deep learning-based approaches [140]. The former includes SAVER [141], SCRABBLE [133], and scRecover [142],which utilize statistical models built on existing data to predict missing data. However, biased results likely arise if the underlying assumptions of the statistic models are not met. On the contrary, deep learning-based methods train neural networks to “learn” the data patterns and their interconnected relationships, minimizing subjective biases. However, they require substantial computational resources, particularly for large datasets [143].

Limited biological understanding of gene expressions presents another challenge for TME characterization. Without sufficient biological knowledge, transient or low biological signals are easily confused with the background noise or artifacts, reducing the signal-to-noise ratio, thus misguiding the identification of true cell phenotypes. Moreover, the presence of specific stimuli and post-translational modifications further hinder the accurate classification of cell phenotypes. A comprehensive understanding of cellular features at single-cell multi-omics levels holds promise for improving the current situation.

Current scRNA-seq technologies are mostly performed on dissociated tumor samples, diminishing the spatial context of each cell, thus emphasizing the need to preserve cellular spatial coordinates in situ. Fluorescent in situ hybridization (FISH)-based methods such as fluorescent in situ sequencing (FISSEQ) [144], starMAP [145], and MERFISH [146] allow for the in situ sequencing of ten to thousands of RNA transcripts. However, this approach proposes a few limitations. First, supervised RNA sequencing requires prior selection of transcripts of interest, compromising the unbiased nature of scRNA-seq. In addition, the measurement of a limited number of RNA transcripts fails to represent the entire transcriptome. Alternatively, separate performances of RNA sequencing and spatial imaging help preserve and combine the strengths of both methods. Strict alignment of tumor dissociates with whole-slide tissue samples is required for generating comparable expressions at gene and protein levels.

### 5.2. Challenges Faced by Cell–Cell Interaction Analysis via Single-Cell Technologies

Current CCC inference tools mostly rely on transcriptomic data, whereas LR interactions primarily occur at the protein level. Transcriptomic analyses are unable to truly reflect protein expressions due to the post-transcriptional and post-translational modifications, likely leading to an inaccurate portrayal of the LR landscape [147]. Direct quantification of cellular surface proteins using proteomic flow cytometry and mass spectrometry technologies helps generate more promising results, while RNA measurements may be used complementarily to increase predicting confidence. An example refers to glycosylation, which alters the structure of 80% of proteins following translation [148]. The glycosylated Asn162 site on the FcγRIIIα receptor was reported with reduced affinities toward fucosylated antibodies, resulting in the lowered interaction strength that crucially influences the antibody-dependent cellular cytotoxicity.

While cell–cell interactions within species have gained heavy attention, those occurring at the interspecies level, particularly between pathogens and human host cells in infectious diseases, require more investigation. Incomprehensive curation of protein–protein interaction databases between pathogens and host cells remains the major limitation to achieving accurate inference [122]. The exponential growth of newly generated data has greatly improved the database scope. Moreover, since pathogens tend to rewire host metabolism for their own survival, the integration of metabolomics information with scRNA-seq data thus provides information with additional biological relevance [149].

Another limitation of CCC inference tools is their failure to account for cellular localization, as only those in proximity enable LR interactions. The introduction of spatial transcriptomic and proteomic technologies, or direct LR screening, which preserves the in situ cellular localization, provides validation of the gene-based inference [150,151,152]. Alternatively, whole-tissue scRNA-seq methods such as PIC-seq mildly dissociate the tumor samples while preserving in situ intracellular interactions to concurrently generate transcriptomic and spatial information [153]. However, only two fluorescent markers are currently available in PIC-seq. Thus, increasing the available markers would help it generate more promising results.

### 5.3. Challenges of Integrating Diverse Single-Cell Datasets

The complex and dynamic biological processes necessitate appropriate integration of single-cell data obtained from different samples, experiments, and measurement types. However, such integration is challenging due to the requirement of flexible and robust statistical models to extract relevant and meaningful biological information from various sources with both accuracy and comprehensiveness. One challenge refers to the batch effects that arise when samples from diverse sources, such as varying time points, tissues, locations, and organisms, are pooled together to analyze similar patterns or differentially expressed genes. Batch effects can also be exacerbated by technical variability including the use of different experimental protocols and sequencing platforms. Standardized experimental protocols, as well as statistical algorithms embedded within the bioinformatics packages, such as Harmony and Scanorama, have been employed to minimize batch effects. Moreover, the creation and constant update of commonly used reference databases, such as the Human Cell Atlas, aids in cell classification and annotation that further support batch effect correction [154].

Another challenge involves integrating multiple “omics”, such as genomic, proteomic, and epigenomic technologies, owing to the distinctive analysis strategies used in each modality. With the interconnections between DNA, RNA, and proteins, many chains of events are identified to form a comprehensive molecular network, providing increasingly reliable reflection of the biological activities. However, single-cell data inherently exhibit sparsity characterized by numerous drop-out events [155]. These missing values are usually estimated by “borrowing” information from other cells. Likewise, noise or sparsity occurring within one modality may be compensated by “borrowing” information from another modality. Over-simplification of the intermodality relationships stems from an incomplete understanding of gene regulatory networks [156]. For example, mRNA-encoded functions may greatly diverge from the actual protein-executed functions owing to post-transcriptional and post-translational modifications. Therefore, there is a critical need to advance our understanding of the underlying biological mechanisms within each modality, along with their temporal dynamics.

### 5.4. Outlook: Translational Insights of Single-Cell Technologies in Clinical Application

The discovery of unique biological features from high-dimensional single-cell data facilitates the creation of low-dimensional gene and/or protein panels suitable for clinical practices. For instance, refined cell types from single-cell transcriptomic analysis can be mapped to bulk RNA-seq samples using bioinformatics methods, allowing for the rapid estimation of cellular compositions in routine clinical samples. Furthermore, protein markers identified through single-cell analysis can be applied to clinically accessible platforms, such as multiplex immunohistochemistry, enabling effective patient stratification based on the molecular features of tumor samples in clinical settings.

Furthermore, the results from single-cell technologies contribute to the curation of public datasets such as the Human Tumor Atlas Network (HTAN), enhancing their utility in clinical settings [157]. The translational potential of HTAN is multi-faceted. Firstly, it aids drug discovery by improving our understanding of drug efficacy and resistance mechanisms. In addition, it enhances diagnostic capabilities by identifying specific cell types associated with early disease development. Moreover, it aids in identifying biomarkers for stratifying patients who likely respond to or resist the therapy. Ultimately, the development of HTAN holds promise for advancing precision medicine, thus improving patient survival outcomes.

## 6. Conclusions

Here, we summarized the bioinformatics tools and analytical steps that are extensively employed in single-cell data analysis in the fields of cancer and immunology. The recently developed single-cell technologies, the subsequent bioinformatics tools, and their applications in the field of cancer treatments have shed light on the underlying mechanisms of immune response against tumor progression. A wide range of studies focusing on various cancer types indicate the potential of single-cell technologies to revolutionize cancer diagnosis in terms of patient stratifications, outcome prediction, treatment options, and long-term monitoring. However, several challenges still remain that remind researchers of the potential biases generated from their results. The future directions of these technologies are briefly discussed, and solutions for improving technological effectiveness, efficiency, and financial cost are proposed.

## Figures and Tables

**Table 1:** Application of single-cell technologies and bioinformatics analysis of different cancer types.

Cancer Type	Single-Cell Technique(s)	Platform(s)	Analysis Tool(s)	Key Finding(s)	Reference(s)
Breast	scRNA-seq	Smart-Seq2	Seurat	Micrometastases display an upregulated OXPHOS pathway with elevated levels of promoting metabolites compared to primary tumors. OXPHOS inhibition greatly attenuates metastasis in lung cancer models, potentiating its value in breast cancer.	[25]
Melanoma	scRNA-seq	10x Genomics Chromium	Seurat	*CCR7*-*SELL*-CD4+ T cell subtype strongly correlates to developing severe immune-related adverse events.	[26]
Prostate	Spatial transcriptomic	Slide-seq	Conos	Stromal cells, including endothelial cells and pericytes, are more chaotically aligned in cancerous prostate; interactions between IGF1+ fibroblasts and IGF1R+ tumor cells are suggested by their colocalization.	[27]
Head and neck	Spatial transcriptomic and proteomic	10x Genomics Visium	Seurat	Two distinct tumor phenotypes were identified: one situated at the leading edge characterized by an accumulation of highly proliferative tumor cells, and the other located at the core with a high infiltration of immune cells.	[28]
Colon	scRNA-seq; Spatial proteomic	10x Genomics Chromium; PenoCycler	Seurat; CACTI	Single-cell analysis discovered the interactions occurring between SPP1+ macrophages and cancer-associated fibroblasts (CAFs) which are high in integrin receptors; spatial analysis demonstrates the spatial proximity between CAFs and SPP1+ macrophages, further suggesting their interactions.	[29]
Lung	scRNA-seq; Spatial transcriptomic	10x Genomics Chromium; 10x Genomics Visium	Seurat	Four cancer subpopulations were identified. The UBE2C+ cell type is strongly correlated with the invasion of lung adenocarcinoma, reflected by its constant increase during the invasion process. The UBE2C+ subpopulation is predominantly located within the peritumoral region and is associated with highly active tumor activities.	[30]

**Table 2:** A summary of scRNAseq analysis steps with corresponding analysis tools and functions.

scRNA-seq Data Analysis Step	Method	Package(s)	Advantage(s)	Limitation(s)	Example Study	Platform	Ref.
Quality control	Ambient RNA removal	SoupX, CellBender	SoupX has a low computational cost. SoupX offers automated estimation of contamination. CellBender allows for simultaneous cell type identification and contamination removal.	SoupX requires prior knowledge on the RNA markers. CellBender leads to ambiguous signal-noise deconvolution if UMI counts within empty droplets are not significantly lower.	[48,49]	R (SoupX) and Terra (CellRender)	[50,51]
	Doublet removal	scDblFinder	High doublet detection accuracy; Computationally efficient.	Detection accuracy reduces with increasing batch.	[52]	R	[53]
Normalization	Scaling-based	sctransform	Uses model-based approach to estimate size factor. Prevents data overfitting through regularization.	Unsuitable for complex data due to assumption of linearity. May bias toward low gene count and zero inflation.	[54]	R	[55]
	Regression-based	SCnorm	Able to scale gene counts between different conditions. Robust for lowly or moderately expressed genes.	High computational cost due to iterative optimization for scaling factor estimation.	[56]	R	[57]
	Spike-in RNA-based	BASiCS	Measures the technical variations and biological heterogeneity simultaneously.	Difference in spike-in and endogenous RNA transcripts may confound the results.	[58]	R	[59]
Confounder correction	Technical-based	scVI, Harmony	Corrects technical artefacts, e.g., batch effects and drop-out events.	Lacks the ability to correct for biological confounders.	[60,61]	R (Harmony) and Python (scVI and scGen)	[62,63]
	Biological-based	Seurat or Scanpy build-ins, Tricycle	Corrects biological confounding features, e.g., low-quality cells, mitochondrial content, and cell cycle effects.	Lacks the ability to correct for technical confounders.	[64]	R (Seurat) and Python (Scanpy and Tricycle)	[46,65,66]
Feature selection	Deviance-based	sctransform	Selects both highly variable and highly expressed gene features. Not effected by normalization due to performance on raw UMI counts.	Highly demanding for memory when scaling large datasets (over 1 million cells).	[67]	R	[55]
	Highly variable gene-based	Seurat	Straight-forward and reduce the risk of losing interesting biological signals.	Performance on normalized gene counts makes it sensitive to normalization.	[68]	R	[65]
	Highly expressed gene-based	Monocle	Simple approach and practical to avoid exhaustive memory runtime.	Performance on normalized gene counts makes it sensitive to normalization. May detect less relevant genes that have high but constant expression across gene set.	[69]	R	[70]
Dimensionality reduction	PCA	Seurat, Scanpy	Preserves the principal components that highly reflect variations across the dataset.	Leads to the loss of underlying biological features stored in the complex data structure.	[71]	R, Python	[46,65]
	t-SNE	Seurat, Scanpy	Models and preserves the non-linear data structure.	Computationally expensive and unable to preserve the global data structure.	[72]	R, Python	[46,65]
	UMAP	Seurat, Scanpy	Computationally efficient with high-dimensional large datasets. Preserves the global data structure.	Potential of data overfitting due to over- emphasis on local structure or noise.	[73]	R, Python	[46,65]
Clustering	Louvain	Seurat, Scanpy	Detects communities with optimal modularity in small to medium-scale datasets; Computationally efficient.	Communities detected may have poor or absent internal connections.	[74]	R, Python	[46,65]
	Leiden	Seurat, Scanpy	Improved version of Louvain; computationally efficient. Guarantees intracommunity connection. Offers optimal local connectivity structure.	Extra phase required to refine the partition.	[75]	R, Python	[46,65]
Annotation	Classifier-based	CellTypist, Clustifyr	Allows for the identification of new genes or cell types. Can be performed on multiple studies.	Requires careful selection of training data. Annotation quality effected by batch effects.	[76,77]	Python, R	[78,79]
	Reference-based	Azimuth, SingleR	Allows for the accurate identification of known markers.	Heavily dependent on prior knowledge. Unable to identify novel markers or cell types.	[26,80]	R, Python	[81,82]

Abbreviations: scRNA-seq—single-cell RNA sequencing; PCA—principal component analysis; t-SNE—t-distributed stochastic neighbor embedding; UMAP—uniform manifold approximation and projection.

**Table 3:** A summary of the LR-based CCC inference tools with a method overview, platforms, and example studies.

CCC Analysis Tool	Approach	Advantage	Limitation	Platform	Example Study	Ref.
iTALK	Identifies general interaction patterns between different cell types based on gene expression profiles.	Enables the prediction of interactions from multiple samples.	The scoring scheme accounts for only abundant genes and thus likely overlooks interactions between less abundant genes.	R	[108]	[101]
PyMINEr	Integrates multi-omics data to identify activating and inhibitory interactions; primarily focuses on identifying metabolic pathways.	Offers the full pipeline from clustering to visualization; there is no requirement for a reference database due to automatic generation; provides additional information on activator and inhibitor.	Signaling pathway-based CCC inference may lead to false prediction due to poor understanding of pathway components, increasing false positive rates.	Python	[109]	[103]
CellPhoneDB	Calculates the permutation-based LR scores to identify significantly up- and downregulated interactions.	Gives strengthened inflammation- and proliferation-related gene sets; reduces false positive rates by using heteromeric complex-based inference.	Shortened in epithelial–mesenchymal transition gene sets; may increase false negative rates.	Python; Python web interface	[110]	[104]
CellChat	Computes the communication score of each LR pair’s interaction strength that are permuted to identify significant interactions.	Reduces false positive rates by using heteromeric complex-based inference; integrates information of mediators and influencers; enables CCC inference in continuous cell states.	Lacks predictions within cell groups; pairwise comparisons limit analysis under different conditions.	R	[111]	[105]
ICELLNET	Calculates both individual and global communication scores to assess cellular communications among single cells or cell types of interests.	Particularly useful in predicting cytokine interactions; enables the incorporation of gene expressions from different datasets; implements experimental validation of the predicted interactions.	Comprises less interactions in the database; lacks information of signaling pathways and gene regulatory networks.	R	[106]	[106]
SingleCellSignalR	Uses regularized product scores to stabilize the noise and variability present in the dataset.	The regularized product score generates a stable threshold to reduce false positive rates.	Unable to integrate information from multiple samples.	R	[112]	[107]
